# Post-Biopsy Cell-Free DNA From Blood: An Open Window on Primary Prostate Cancer Genetics and Biology

**DOI:** 10.3389/fonc.2021.654140

**Published:** 2021-05-24

**Authors:** Marinella Corbetta, Chiara Chiereghin, Ilaria De Simone, Giulia Soldà, Monica Zuradelli, Michele Giunta, Giovanni Lughezzani, Nicolò Maria Buffi, Rodolfo Hurle, Alberto Saita, Paolo Casale, Rosanna Asselta, Massimo Lazzeri, Giorgio Guazzoni, Stefano Duga

**Affiliations:** ^1^ Department of Biomedical Sciences, Humanitas University, Milan, Italy; ^2^ IRCCS Humanitas Research Hospital, Milan, Italy; ^3^ IRCCS Humanitas Research Hospital, Humanitas Cancer Center, Milan, Italy

**Keywords:** circulating cell-free DNA, circulating tumor DNA, primary prostate cancer, biopsy, biomarker

## Abstract

Circulating cell-free DNA (ccfDNA), released from normal and cancerous cells, is a promising biomarker for cancer detection as in neoplastic patients it is enriched in tumor-derived DNA (ctDNA). ctDNA contains cancer-specific mutations and epigenetic modifications, which can have diagnostic/prognostic value. However, in primary tumors, and in particular in localized prostate cancer (PCa), the fraction of ctDNA is very low and conventional strategies to study ccfDNA are unsuccessful. Here we demonstrate that prostate biopsy, by causing multiple injuries to the organ, leads to a significant increase in plasma concentration of ccfDNA (P<0.0024) in primary PCa patients. By calculating the minor allele fraction at patient-specific somatic mutations pre- and post-biopsy, we show that ctDNA is significantly enriched (from 3.9 to 164 fold) after biopsy, representing a transient “molecular window” to access and analyze ctDNA. Moreover, we show that newly released ccfDNA contains a larger fraction of di-, tri- and multi-nucleosome associated DNA fragments. This feature could be exploited to further enrich prostate-derived ccfDNA and to analyze epigenetic markers. Our data represent a proof-of-concept that liquid tumor profiling from peripheral blood performed just after the biopsy procedure can open a “valuable molecular metastatic window” giving access to the tumor genetic asset, thus providing an opportunity for early cancer detection and individual genomic profiling in the view of PCa precision medicine.

## Introduction

The analysis of circulating cell-free DNA (ccfDNA) represents one of the most promising strategies for non-invasive disease monitoring (1). In cancer management, it has been effectively used to follow tumor evolution and acquisition of resistance, as well as for guiding treatment decisions in multiple non-hematologic diseases. ccfDNA is thought to be released into the bloodstream by all cells, both by active secretion and as a consequence of death by apoptosis or necrosis, in response to both physiological processes (i.e. exercise), as well as malignant and non-malignant pathological conditions, such as inflammation and tissue damage (2). Although the first description of ccfDNA in plasma dates back to 1948 (3), abnormalities in patients with cancer were observed only decades later (4, 5), and the exact mechanism of its release and its biological properties still remain unclear.

During cancer development, tumor-derived DNA is released in the circulation (circulating tumor DNA, ctDNA) since early stages of the disease and mixes with a larger amount of physiologically released nonmalignant DNA, which is mostly derived from hematopoietic cells (6, 7). The fraction of cancer-derived ccfDNA is highly variable, and mainly depends on two factors: i) the advancement of the disease, with more ctDNA reflecting a greater disease burden, and ii) the location of the tumor, with colon, gastroduodenal tract, breast, pancreas, liver, and skin cancers releasing large amounts of ctDNA, whereas glioma, thyroid, kidney, and prostate tumors are associated with the smallest amount of ctDNA in plasma (8, 9). These tissue differences have been explained by the presence of the blood-brain barrier for gliomas and of an organ capsule/pseudocapsule for thyroid, prostate, and kidney cancer, limiting the diffusion of ctDNA into blood (9).

Once released in the circulation, ccfDNA undergoes a rapid degradation by nucleases (10, 11), with an estimated half-life of fewer than 2 h (12, 13). Therefore, most ccfDNA presents as a 160- to 170-bp peak, roughly corresponding to the portion of DNA protected by the interaction with histones in nucleosomes (14, 15). Larger fragments, of about 360 to 400 bp and more, have also been reported, likely representing multimers of nucleosomes (16). Dying cells and tumors undergoing cell necrosis were shown to release additional longer fragments, up to 10 kb (14). Tissue-specific methylation signatures and nucleosome phasing analysis have been effectively used to derive the organ of origin of ccfDNA (7, 15, 17). Recently, the fragment length of ctDNA from different cancers was found to be more variable compared to non-cancer ccfDNA, probably reflecting tumor-specific changes in chromatin structure and epigenetics marks, suggesting that ccfDNA fragmentation could serve as a biomarker for cancer detection (18).

Concerning prostate cancer (PCa), only few studies have explored ctDNA as a potential biomarker in localized disease to distinguish PCa from benign prostatic hyperplasia in patients with elevated prostate-specific antigen (PSA) (19), to provide prognostic information in patients undergoing radical prostatectomy (20), or to follow-up patients with PCa (21). More recently, Hennigan and colleagues confirmed that in localized PCa, allele-specific alterations in ctDNA are below the threshold for detection, even in high-risk patients who will eventually develop disease recurrence (22). A larger number of studies, conversely, explored the feasibility to use ctDNA in advanced metastatic and castration-resistant disease, demonstrating that ctDNA from liquid biopsies can be used to get insights into the mutational burden of metastatic PCa, without the need for direct tissue sampling, and as prognostic biomarker of response to therapy (23–29).

It has been clearly demonstrated that certain conditions, including inflammation, exercise, or tissue injury, substantially increase the ccfDNA level. In particular, it was shown that ccfDNA levels may increase by more than an order of magnitude during surgery (12). Similarly, we could hypothesize that prostate biopsy, by requiring multiple punctures of the organ, would release a large amount of prostate DNA representative of all analyzed regions, creating a sort of temporary time window for ccfDNA analysis, which may allow getting genetic information for a precision-decision making, even in localized PCa. Hence, we tested the hypothesis that prostate biopsy may generate a transient increase in prostate-derived ccfDNA, which is also likely to be enriched in ctDNA, and could provide molecular data on the tumor of diagnostic, prognostic, and theranostic relevance.

## Materials and Methods

### Study Cohort and Sample Collection

The study was approved by the Humanitas Clinical and Research Center Ethics Committee (336/19) and all participants signed an appropriate informed consent. We enrolled 38 patients who underwent prostate biopsy for suspected PCa according to indications from their referring urologists. Twenty-one patients already had a prostate biopsy in the past that detected PCa Gleason 3 + 3 in 10 and 3 + 4 in 1 patient. Inclusion criteria were the following: i) patients >45 years of age with or without a positive digital rectal examination (DRE); ii) total PSA >2 ng/ml, according to Hybritech B&C calibration and suspected mpMRI for PCa (Prostate Imaging-Recording and Data System [PI-RADS] v.2 – score >=3). Patients with a previous diagnosis of PCa who were under active surveillance and high-grade prostatic intraepithelial neoplasia or atypical small acinar proliferation were also considered as cases. Exclusion criteria were the following: i) patients with bacterial acute prostatitis in the 3 months prior to biopsy; ii) patients subjected to previous endoscopic surgery of the prostate; iii) patients being treated with dutasteride or finasteride. Subjects with chronic renal failure, marked blood protein alterations (plasma normal range, 6–8 g/100 ml), hemophiliacs, or those previously submitted to multiple blood transfusions were not included in the study, as these conditions may alter the analysis. mpMRI/TRUS software assisted fusion-guided biopsy was performed in 31 patients by two experienced urologists (ML and GL) with more than 500 procedure each one. The prostate profile and regions of interest (ROIs) were manually contoured by the expert urologists and radiologists. Images of the prostate and mpMRI derived ROIs were fused in real time for biopsy. The procedures were performed by transperineal or transrectal approach and two or more cores for single ROI were sampled plus a systematic random biopsy (12 cores) according to EAU Guideline for biopsy-naïve patients. Seven patients received a trans rectal ultrasound guided systematic (12 core) biopsy. In patients whose biopsies were studied by RNA sequencing, the analyzed core was harvested from the highest PIRADS ROI or mpMRI index lesion.

After the diagnosis of PCa, 18 patients underwent radical prostatectomy, including two patients under active surveillance (AS) who were up-graded to clinical significant PCa. Four patients under AS, who were upgraded, opted for androgen deprivation therapy or radiotherapy and seven continued their follow-up according to Prostate Cancer Research International Active Surveillance (PRIAS) protocol. One patient with a very high risk disease received radiotherapy and ADT. Five patients who presented benign prostate hyperplasia received a medical therapy. Three patients were referred to their general practitioner.

Fresh-needle biopsies and blood samples were collected from all 38 patients, whose clinicopathological information are summed up in **Table 1**. Fresh-needle biopsies were immersed into the RNAlater stabilization solution (Sigma-Aldrich, St Louis, MO, USA) immediately after withdrawal and were left overnight at 4°C to allow the solution enter the sample. After RNAlater removal, biopsies were stored at −80°C. Blood samples, approximately 3 ml each, were collected in Vacutainer^®^ EDTA tubes and withdrawn from patients prior and after biopsy, at different time points (10, 30, 60, and 120 minutes after the procedure).

**Table 1:** Patient characteristics and biopsy results.

Status*	N	Age, years (mean ± SD; range)	Pre-biopsy PSA level, ng/mL (median; [IQR])	% positive cores (mean ± SD; range)	Prostate volume, mm^3^ (mean ± SD; range)
**No cancer**	5	67 ± 10; 52–76	6.00 [3.97–11.62]	0	n.a.
**3+3**	7	65 ± 5; 57–71	5.40 [5.10–10.55]	14.8 ± 20.4; 12.5–57.1	40.3 ± 15.9; 22–49.8
**3+4**	13	67 ± 6; 51–74	8.20 [5.89–12.90]	31.8 ± 17.9; 15.8–60	33.2 ± 19.7; 12.48–60
**4+3**	5	72 ± 4; 69–78	9.96 [5.25–17.80]	36.1 ± 25.2; 18.8–80	42.7 ± 18; 31.8–63.4
**4+4**	6	63 ± 7; 55–70	13.54 [10.67–24.48]	68 ± 39; 6.3–100	68.6 ± 3.67; 66–72.8
**4+5**	1	56	23.14	100	n.a.
**5+5**	1	80	20	58.3	n.a.

Pre-biopsy PSA level is indicated as median and interquartile range (IQR) expressed as [quartile 1- quartile 3]. All other parameters are indicated as mean ± standard deviation (SD).

N, number; n.a., not available; PSA, prostate-specific antigen.

*Patients with PCa were classified according to the Gleason Score.

### Isolation of ccfDNA

Isolation of ccfDNA was performed within 1 hour from blood withdrawal; samples were centrifuged to isolate plasma for 10 minutes at 2000g at room temperature. To ensure that the cellular component was completely removed, plasma was centrifuged again at 2000g for 10 minutes at room temperature. The collected plasma was either immediately processed for ccfDNA extraction with the Maxwell RSC ccfDNA Plasma Kit (Promega, Madison, WI, USA) using the Maxwell instrument (Promega), or stored at −20°C until use. DNA was quantified using a fluorometer (Thermo Fisher Scientific, Waltham, MA, USA) with the Qubit™ dsDNA HS Assay Kit (Thermo Fisher Scientific). Qualitative analysis was performed with the Agilent High Sensitivity D5000 ScreenTape System on Agilent-4200 TapeStation (Agilent Technologies; Santa Clara, CA, USA).

### RNA Extraction From Biopsies

Molecular analyses were performed on a total of 28 patients. Biopsy samples were disrupted and homogenized in presence of Tungsten Carbide Beads, 3 mm diameter (QIAGEN, Hilden, Germany) with the TissueLyser II (QIAGEN). RNA was extracted from the homogenized samples using the Maxwell^®^ RSC miRNA Tissue Kit, with the automated Maxwell instrument, according to the manufacturer’s protocol. RNA was quantified by Qubit fluorometer with Qubit™ RNA HS Assay Kit (Thermo Fisher Scientific) and RNA quality was assessed with the Agilent High Sensitivity RNA Screen Tape (Agilent Technologies) on an Agilent-4200 Tapestation, obtaining a mean RNA integrity number (RIN) value of 7.05 (max value 10, min value 3.7).

### Next-Generation Sequencing (NGS) Analysis

Targeted RNA sequencing was performed using the TruSight RNA Pan-Cancer Panel (Illumina, San Diego, CA, USA). Libraries were prepared starting from 55 to 75 ng of RNA, following the manufacturer’s protocol. cDNA libraries, synthesized from fragmented RNA, were sequenced as 76-bp paired-end reads on a NextSeq550 platform (Illumina).

Sequencing data were analyzed using the RNAseq alignment v. 2.0.10 pipeline on BaseSpace (Illumina). Briefly, input reads were filtered against abundant sequences, such as mitochondrial or ribosomal sequences, using Bowtie 0.12.9 (30), and then aligned to the reference human genome (UCSC hg19) and the RefSeq annotation of transcripts using the Spliced Transcripts Alignment to a Reference (STAR) program (v. 2.6.1a) (31). Single nucleotide variants (SNVs) were identified with the Strelka Variant Caller v.2.9.9 (32), and the presence of fusion transcripts was detected with the Manta Structural Variant Caller v.1.4.0 (33).

### Somatic SNV Selection From Patients’ Bioptic Tissue

The variant call lists were filtered to identify somatic SNVs. Five rare variants (with minor allele frequency, MAF, <0.01, for annotated variants) covered by at least 50 reads and falling into exonic regions were selected and verified by Sanger sequencing. For all chosen variants, one primer pair spanning the region containing the SNV of interest was designed. Genomic DNA was extracted from the cellular components of blood samples recovered after plasma separation. 1.5 mL of phosphate buffered saline (PBS) 1x was added to blood cellular components and the extractions were performed from 400 μL of resuspended cells with the Maxwell 16 Blood DNA Purification kit (Promega). PCR reactions were conducted by using the GoTaq^®^ G2 Flexi DNA Polymerase (Promega), according to the manufacturer’s instructions. Sequencing reactions were performed using the BigDye Terminator Cycle Sequencing Ready Reaction Kit v1.1 (Thermo Fisher Scientific) and then loaded on an ABI-3500 sequencer (Thermo Fisher Scientific). Electropherograms were analyzed with the FinchTV software. All primers were purchased from Sigma. Primer sequences and PCR cycling conditions for each amplicon are available on request.

### Detection of Selected Somatic Mutations in ccfDNA

To amplify the genomic regions containing the selected somatic variants in pre- and post-biopsy ccfDNA, PCR assays were designed and performed using the low-error rate Q5^®^ High-Fidelity DNA Polymerase (New England BioLabs, Ipswich, MA, USA). Each reaction was carried out in a final volume of 50 μL, following the manufacturer’s instructions, and starting from at least 1 ng of ccfDNA. For each variant, the same PCR was performed starting from pre- and post-ccfDNA of the relevant patient. Purified PCR products were quantified with the Qubit™ dsDNA HS Assay Kit on the Qubit fluorometer. NGS analysis of amplicons was carried out with the NEBNext^®^ Ultra™ II DNA Library Prep Kit for Illumina (New England BioLabs). In this regard, two different amplicon pools were generated: a pre-biopsy ccfDNA and a post-biopsy ccfDNA amplicon pool; in each pool amplicons were equally represented. Libraries were prepared following the manufacturer’s instructions, starting from the same amount of material for each coupled pre- and post-biopsy pool, varying from a minimum of 195 ng to a maximum of 490 ng. Libraries were sequenced on the NextSeq550 platform obtaining a mean depth of 235,367x. Read number and base count of each variant were reported following BAM alignment on the hg19 reference genome, through visualization with the Integrative Genomics Viewer tool (34).

### Statistical Analysis

All statistical analyses were performed using GraphPad Prism version 7.01 for Windows (GraphPad software, San Diego, California USA, www.graphpad.com). Wilcoxon matched-pairs signed rank test was applied to calculate statistical significance of the observed differences in ccfDNA levels between pre- and post-biopsy conditions at all analyzed time points. The same statistical test was also used to evaluate the size increase of ccfDNA peaks pre- and post-biopsy.

## Results

### Clinical and Histological Features of Analyzed Subjects

A total of 38 patients referred to prostate biopsy for suspected PCa were enrolled in the study (**Table 1**). The median preoperative PSA of these patients was 9.96 ng/mL [IQR 5.35 ng/mL to 17.8 ng/mL]. Upon biopsy analysis, 5 patients had no evidence of PCa, 7 had lSUP1 (Gleason score <3+4), 18 had ISUP2 or 3 (Gleason score 3 + 4 and 4 + 3, respectively), and 8 had ISUP4 or 5 (Gleason score >4+3).

### Prostate Biopsy Increases ccfDNA Levels in Blood

A first blood sample for ccfDNA extraction was taken from 32 patients immediately before the biopsy procedure. A second blood sample was collected either 60 (N=13) or 120 (N=19) minutes after the biopsy. The amount of ccfDNA extracted from 1 mL of plasma was measured using a QubitVR 2.0 Fluorometer.

**Figure 1:**
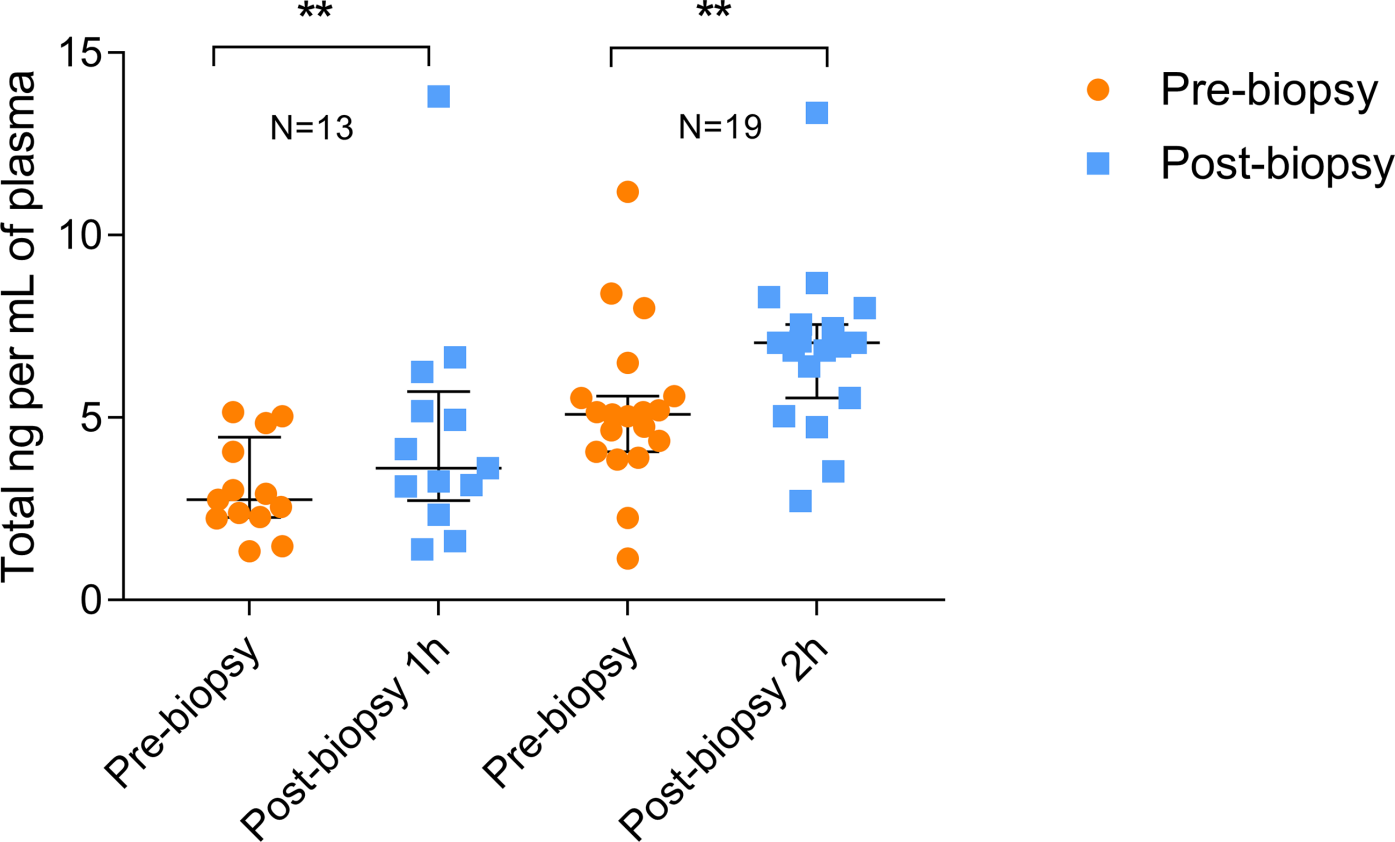
Quantitation of pre- and post-biopsy ccfDNA from blood. ccfDNA extracted from blood immediately before the biopsy and after 1 or 2 hours from the end of the procedure. Scatter plots (with median and interquartile range) show the amount of ccfDNA expressed as ng/mL of plasma. The number of analyzed individuals in shown above the plots (** P<0.01; Wilcoxon test for paired samples).

As shown in **Figure 1**, the total amount of ccfDNA was significantly increased in the post-biopsy samples compared to pre-biopsy ones at both time points (60 min: pre-biopsy median 2.76 ng/mL, IQR: 2.29–4.07 ng/mL vs post-biopsy median 3.62 ng/mL, IQR: 3.12–5.19 ng/mL, P=0.0024; 120 min: pre-biopsy 5.1 ng/mL, IQR: 4.22–5.58 ng/mL vs post-biopsy, median 7.05 ng/mL, IQR: 5.98–7.5 ng/mL, P=0.0023). No correlation between post-biopsy ccfDNA levels and Gleason score was observed (data not show).

### Kinetics of ccfDNA Increase After Prostate Biopsy

**Figure 2:**
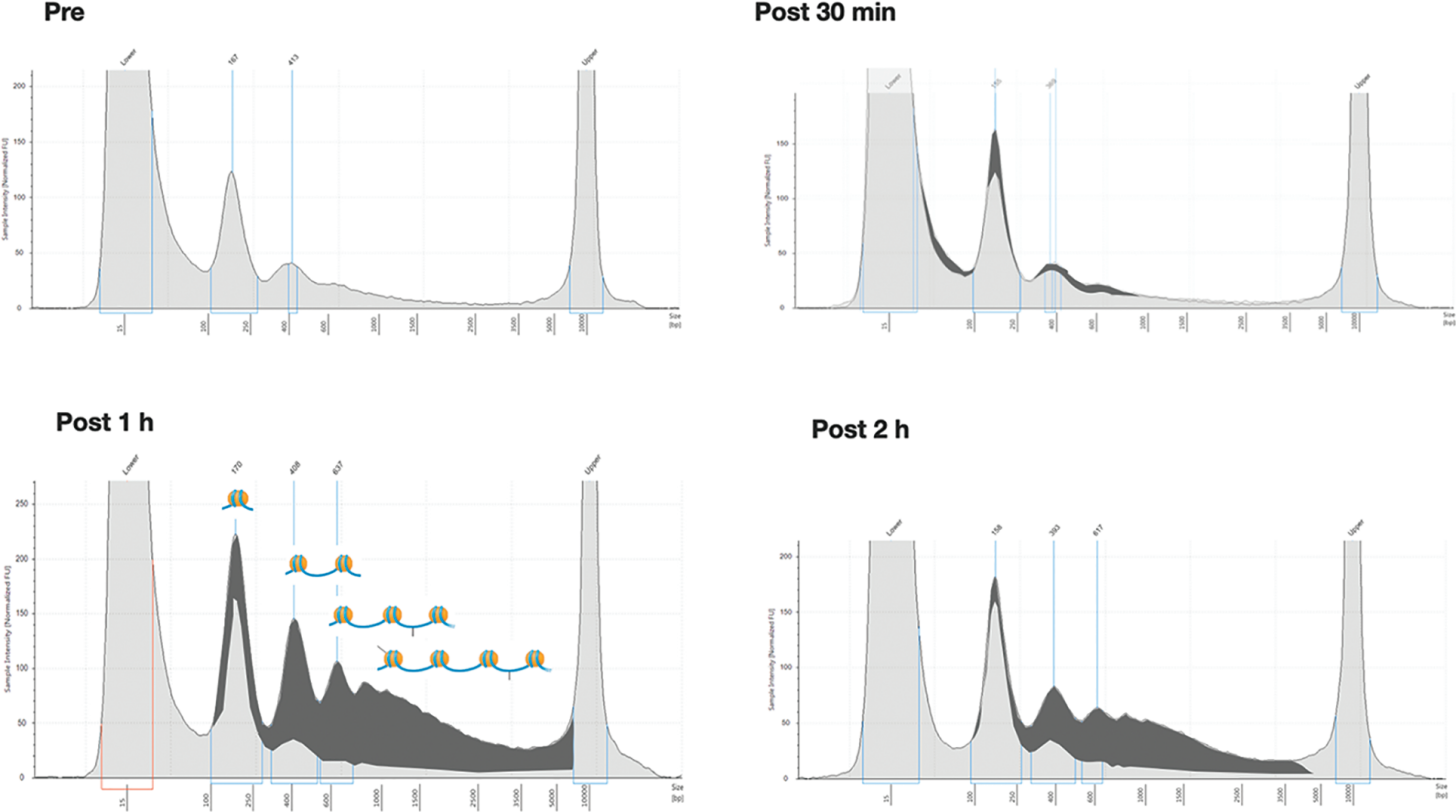
Overall size distribution of ccfDNA pre- and post-biopsy. Representative Tape station electropherograms showing DNA fragment size distribution in ccfDNA isolated from plasma of a single patient (P20) pre-biopsy and after 30 min, 1 hour, and 2 hours from the procedure. The longer fragments appearing after 1 and 2 hours post-biopsy are highlighted in dark grey as the portion of the area under the curve after subtracting the profile obtained for pre-biopsy ccfDNA. In the 1h panel a schematic representation of the predicted nucleosomal structure of the detected fragments is depicted above the profiles.

Six additional patients were analyzed to describe the time course of ccfDNA release after biopsy. For this purpose, in addition to the pre-biopsy sample, a total of 4 blood samples at different time points (10, 30, 60, and 120 min after the procedure) were taken for each patient. The extracted ccfDNA was then analyzed for size and concentration. Comparing the profiles of each patient at different time points (see as an example the quali/quantitative reports in **Figure 2**) we could confirm a significant increase in the size of ccfDNA fragments after the biopsy. In particular, at both 60 and 120 minutes after biopsy, we observed the presence of a significant amount of ccfDNA fragments longer than 350 bp (up to 1.5 kb), likely corresponding to DNA associated to multiple nucleosomes (**Figure 2****)**.

**Figure 3:**
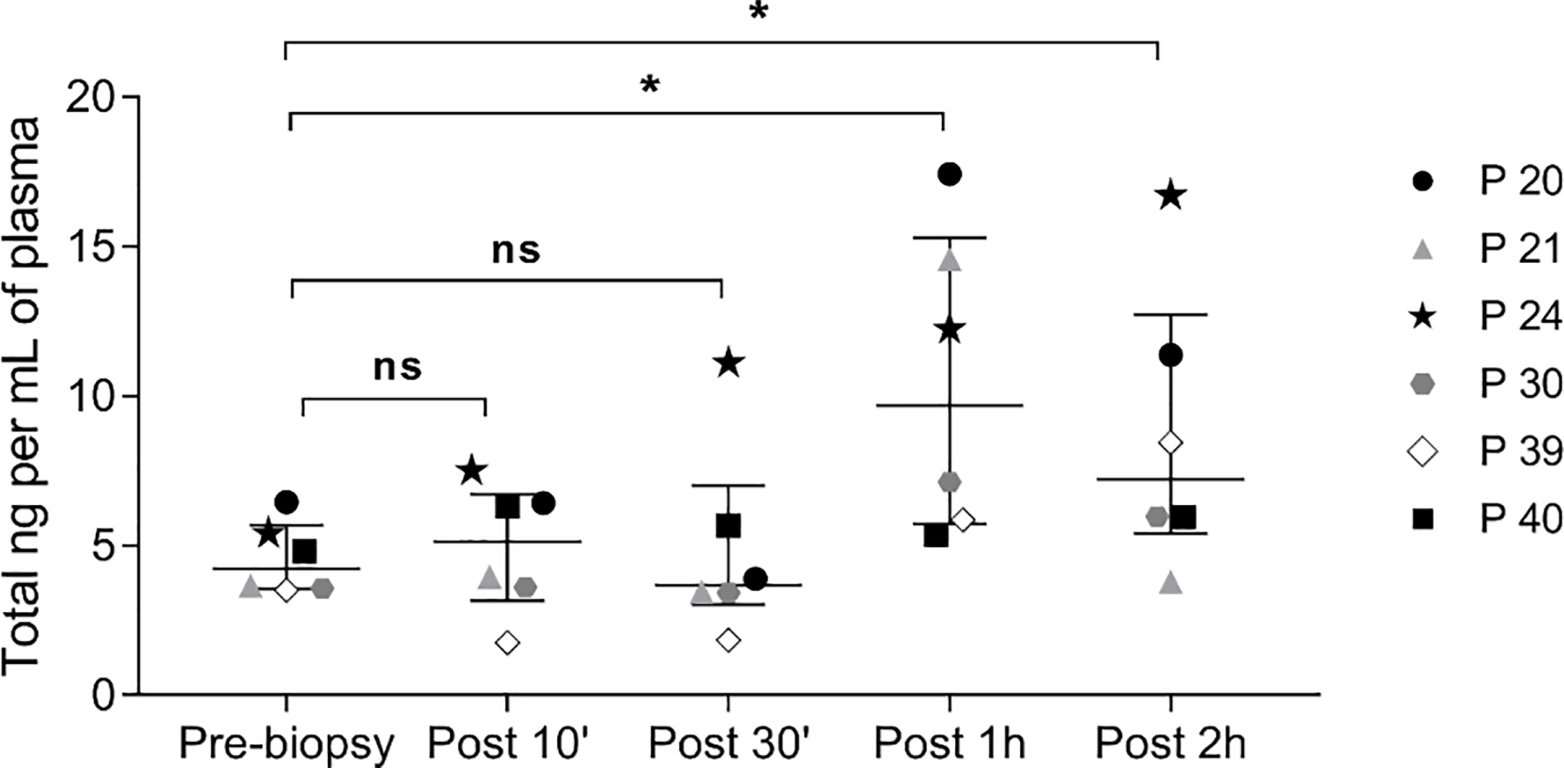
Kinetics of ccfDNA release in circulation after prostate biopsy. Scatter plots, with median and interquartile range, of ccfDNA concentration (ng/mL plasma) as quantified by the Qubit fluorometer in the plasma of 6 patients at different time points. ns, not significant; * P< 0.05; Wilcoxon test for paired samples.

Moreover, in all six cases, the kinetics of ccfDNA release in circulation showed an increase in ccfDNA levels with time, with the highest average ccfDNA concentration observed 1 hour after the procedure; however, only some patients showed a peak in ccfDNA at 1 hour, while in others the increase continued at 2 hours post biopsy (**Figure 3**).

### Size Distribution Analysis of ccfDNA

**Figure 4:**
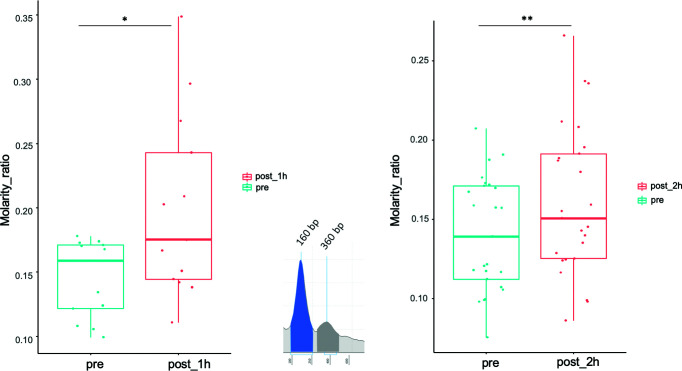
Size shift in post-biopsy ccfDNA vs. pre-biopsy ccfDNA. Boxplots show the molarity ratio between the 360 bp (DNA associated to two consecutive nucleosomes) and the 160 bp (DNA associated with a single nucleosome) peaks obtained by measuring the peak area, one hour and 2 hours from the biopsy procedure. Boxes define the interquartile range; the central line refers to the median. The number of analyzed individuals in shown below the graph (*P < 0.05, **P < 0.01; Wilcoxon test for paired samples).

In pre-biopsy samples, a typical ccfDNA size-pattern was found, with a main 160 to 165 bp peak and a minor 360 to 400 bp peak, representing mono and di-nucleosomal lengths, respectively (**Figure 4** **)**. Comparing pre- and post-biopsy samples, the increase in the area of the 360-bp peak was significantly higher than the one measured for the 160-bp peak (75.11% vs. 37.46%, P=0.0007), confirming that post-biopsy ccfDNA contains qualitatively different DNA. Moreover, in post-biopsy ccfDNA, additional peaks were visible at about 630 bp and 870 bp, likely representing further nucleosomal multimers, as well as fragments larger than 1 kb (**Figure 4**).

### ctDNA Enrichment in Post-Biopsy ccfDNA

**Figure 5:**
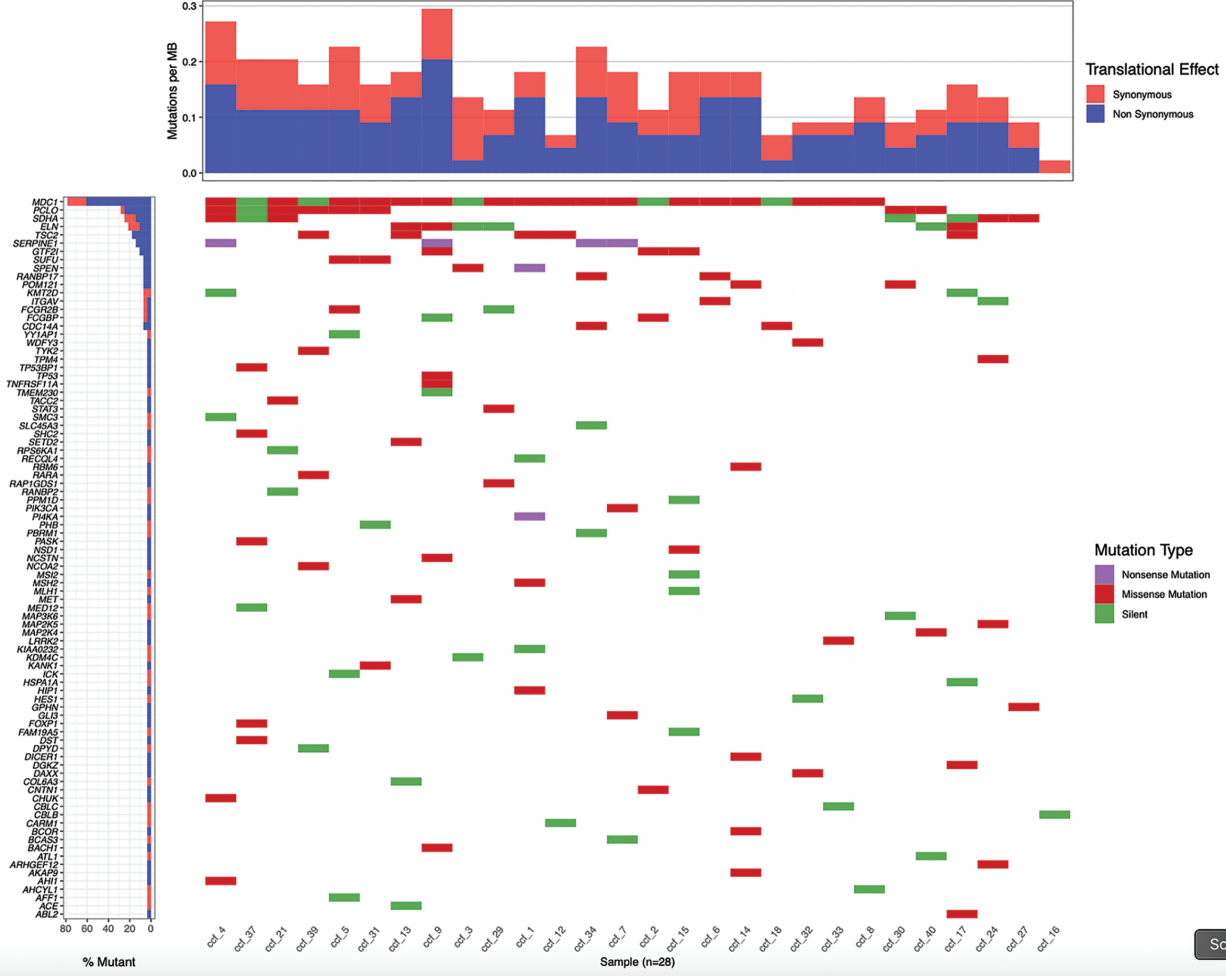
Patient-specific candidate PCa somatic variants from biopsy samples. Waterfall plot representing 188 candidate somatic variants detected in 28 prostate biopsy samples by targeted RNA sequencing. Each column represents a sample and each row a gene. Mutations are colored according to the predicted functional consequence. The plot was generated using the GenVisR Bioconductor package.

The significant increase in ccfDNA after the biopsy is likely to derive from the tissue damage caused by the needle puncture injury. So, we hypothesized that the ccfDNA released in the bloodstream should be enriched in prostate-derived DNA and, particularly, in ctDNA. To validate this hypothesis, we searched for patient-specific somatic mutations in the tumor tissue (obtained from a fresh biopsy of the index lesion) by targeted RNAseq using the TruSight RNA Pan-Cancer kit, which enriches for 1,385 cancer-related genes, including some of the most frequently mutated genes in PCa. A total of 28 patients were selected for molecular analysis. We obtained a mean number of reads per patient of 22.7 M with an average of 98% of aligned reads to the reference genome. Coding regions were covered with an average depth of 721.7×. Variant calling on NGS data found on average 4,112 SNVs per patient. To identify likely somatic variants, we selected only exonic SNVs with an alternative allele fraction ≤0.35, MAF<0.01 in the general population, and covered by at least 50 reads. Variants that were recurrently found in more than 25% of the samples were excluded as putative sequencing artifacts. After prioritization, a total of 188 candidate somatic variants (on average 7 per patient) in 87 genes were left (**Figure 5****)**. Of these, 106 were missense, 76 were synonymous/silent, and 6 nonsense.

Five variants, namely NM_005338.7(HIP1):c.2167G>A(p.Glu723Lys), NM_000546.5 (TP53):c.747G>C/T(p.Arg249Ser), NM_014991.5(WDFY3):c.718A>G(p.Met240Val), NM_022455.5(NSD1):c.3049A>T(p.Thr1017Ala), and NM_015001.3(SPEN): c.2500C>T(p.Gln834Ter), in four patients were selected and validated by Sanger sequencing. Absence in germline DNA confirmed that all selected variants (one nonsense, four missense) were somatic. We then performed tumor-guided personalized deep sequencing in each patient’s ccfDNA, by amplifying the relevant DNA fragment and submitting the pool of amplified fragments to NGS. The number of mutated reads divided by the total reads at each of the mutation sites in post-biopsy ccfDNA was calculated and compared with the same ratio calculated on ccfDNA before the biopsy procedure. In all cases, a dramatic enrichment in ctDNA was found, ranging from 3.9 to 164 times the amount present in circulation before the surgical procedure (**Table 2**).

**Table 2:** Enrichment in ctDNA post biopsy.

Gene	Pre ccfDNA					Post ccfDNA					Fold enrichment			
	A	T	C	G	Tot counts	A	T	C	G	Tot counts	A	T	C	G
***SPEN***	395	56	344,112	71	344,634	384	2,228	366,751	75	369,440	0.9	**37.1**	1.0	1.0
***HIP1***	273	67	514,588	138	515,067	193	9,007	413,019	95	422,316	0.9	**163.9**	1.0	0.8
***TP53***	1.231	36	278,734	162	280,167	2,517	39	263,754	601	266,912	**2.2**	1.1	1.0	**3.9**
***NSD1***	27.761	55	0	7	27,823	23,025	52	0	151	23,228	1.0	1.1		**25.8**
***WDFY3***	15	64,070	8	3	64,096	13	39,801	169	2	39,987	1.4	1.0	**33.9**	1.1

The fold increase of ctDNA was calculated by dividing the proportion of the reads supporting each of the four nucleotide calls on the total number of reads in the post biopsy ccfDNA by the proportion of reads of the same nucleotide in the pre-biopsy sample. Fold enrichments of the analyzed somatic variants are marked in bold.

## Discussion

As the debate continues on the merits of PCa detection, urologists are struggling to balance benefits from early detection and treatment of lethal PCa from overdiagnosis and overtreatment of clinically insignificant PCa. The landscape of PCa early detection approach and management continues to evolve thanks to the understanding of the value of PSA isoforms (35), as well as of other molecular and imaging biomarkers (36). However, there are still many areas of research where efforts to optimize patient selection for diagnosis and risk stratification for treatment are ongoing. The emerging data on somatic and germline mutations in PCa have provided additional insights into the importance of genetic testing to identify clinically significant PCa (37). There is, therefore, a strong unmet clinical need for non-invasive biomarkers in early disease for better stratifying patients with aggressive tumors from those not in need of further intervention. Such an approach should discriminate cancer patients from healthy subjects, distinguish clinically significant from indolent PCa, guide therapy, and predict prognosis and follow-up.

The data currently present in the literature suggest that in patients with primary PCa it is not possible to exploit the analysis of ctDNA as the quantity of ctDNA released into the circulation is insufficient (22). Here we set up and tested an alternative approach, which uses the inherent characteristics/consequences of the prostate biopsy procedure as a way to obtain increased amounts of prostate-derived ccfDNA and, possibly, ctDNA from blood. By measuring the amount of ccfDNA before and after a prostate biopsy (index lesion plus a standard 12-core schema) we were able to describe, for the first time, the size profile and the kinetics of ccfDNA release in circulation after prostate biopsy. Moreover, by looking at patient-specific somatic mutations we showed that post-biopsy ccfDNA is significantly enriched in tumor-derived ctDNA. We found no correlation between post-biopsy ccfDNA levels and Gleason score, which suggests that probably malignancy does not correlate directly with the number of undifferentiated cells. Differences in post-biopsy ccfDNA levels might depend on the level of traumatism induced during the surgical procedure.

As a crucial biological property of ccfDNA, size profile has been assessed by a variety of methods, including gel electrophoresis, atomic force microscopy (AFM), quantitative real-time PCR (qPCR), and NGS (38). In most cases, the size profile of ccfDNA was distributed as a “ladder” pattern with a major peak at ~166 bp. However, by using AFM, Mouliere and colleagues showed that 80% of ccfDNA in colorectal cancer patients is <145 bp (39) and, by qPCR, that the ccfDNA with *KRAS* mutation is more fragmented than the wild-type ccfDNA in colorectal cancer patients (40). Also, by using NGS, it was reported that short ccfDNA fragments preferentially carried the tumor-associated aberrations in hepatocellular carcinoma patients (41). Therefore, tumor ccfDNA is generally considered to be shorter than non-tumor ccfDNA, although some evidence points to the opposite: for example, by NGS, it was reported that the median overall size of ccfDNA in tumor patients is around 163.8 bp (18). Furthermore, the ccfDNA isolated from early stages of pancreatic and breast cancer was observed to be longer than that found in metastatic patients (42, 43). Concerning PCa, it is interesting to note that in seminal fluid fragments longer than 1000 bp were shown to be more abundant than in healthy subjects (44). These discrepancies, besides deriving from the different techniques used to measure the ccfDNA size, may also reflect different subnucleosomal fragmentation patterns of ccfDNA. In particular, hypomethylation of DNA, a frequent finding in cancer, may increase the accessibility of ccfDNA to nucleases, giving reason for the shorter size distribution of ccfDNA in most cancer patients. Our experiments measured ccfDNA immediately after a surgical procedure, which is likely to damage a large number of cells and create the condition for the subsequent death of nearby cells, promoting a release of ccfDNA mainly due to cell break than to apoptotic pathways. This is probably the reason why we could observe a significant increase in the size of ccfDNA, as demonstrated by the increased ratio between the single-nucleosome and the double-nucleosome DNA peaks. This difference in fragment lengths could be exploited to enhance sensitivity for detecting the presence of ctDNA and for noninvasive genomic analysis of cancer by performing a first PCR step with long amplicons, thus selecting longer ccfDNA fragments, more likely to come from the damaged prostate tissue. This would be an approach similar, even though opposite, to the one recently described by Mouliere and colleagues (45). Further studies will evaluate whether, with this enrichment step, it is possible to detect somatic mutations directly from ccfDNA in primary PCa.

The release of ctDNA from the prostate can also be exploited to check for epigenetic modifications, which represent useful markers to improve the stratification of patients with aggressive and indolent PCa (46, 47). In particular, a four-gene prognostic model was derived by analyzing DNA methylation changes in 12 genes associated with disease progression and validated to improve the prediction of recurrence in patients with PCa after surgery (48).

In conclusion, our experiments pave the way to perform ccfDNA analysis also in primary PCa by exploiting the transient release in circulation of prostate ctDNA immediately after the biopsy procedure. This can give easy access to DNA from the entire organ, being superior (and cheaper) compared to the analysis of single biopsies, in the view of the frequent multifocal nature of PCa. ctDNA obtained after biopsy can be studied for the presence of somatic mutations or epigenetic alterations, thus allowing to anticipate a precision medicine-based approach to therapy.

## Data Availability Statement

The datasets presented in this study have been deposited in the Zenodo repository under the accession number: 10.5281/zenodo.4454599. The datasets presented in this article are not readily available because it includes sensitive data. Requests to access the datasets should be directed to the corresponding author.

## Ethics Statement

The study was reviewed and approved by the Humanitas Clinical and Research Center Ethics Committee (336/19). The patients/participants provided their written informed consent to participate in this study.

## Author Contributions

Conception and design: SD, RA, and ML. Acquisition of data: CC, IS, MZ, MG, GL, NMB, RH, AS, and PC. Analysis and interpretation of data: SD, RA, CC, and ML. Drafting of the manuscript: MC. Critical revision of the manuscript for important intellectual content: SD, RA, MZ, ML, GS, and GG. Statistical analysis: RA. Obtaining funding: GG. Administrative, technical, or material support supervision: SD. All authors contributed to the article and approved the submitted version.

## Funding

This work was supported by the Ministero della Salute (Ricerca Finalizzata, grant number RF-2018-12367080) and by intramural funding (Fondazione Humanitas per la Ricerca). We would also like to acknowledge the financial aid of a number of friends who supported our research with personal donations.

## Conflict of Interest

The authors declare that the research was conducted in the absence of any commercial or financial relationships that could be construed as a potential conflict of interest.
